# Combined Intestinal Metabolomics and Microbiota Analysis for Acute Endometritis Induced by Lipopolysaccharide in Mice

**DOI:** 10.3389/fcimb.2021.791373

**Published:** 2021-12-17

**Authors:** Yuqing Dong, Yuan Yuan, Yichuan Ma, Yuanyue Luo, Wenjing Zhou, Xin Deng, Jingyu Pu, Binhong Hu, Songqing Liu

**Affiliations:** ^1^ College of Chemistry and Life Sciences, Chengdu Normal University, Chengdu, China; ^2^ College of Forestry, Sichuan Agricultural University, Chengdu, China; ^3^ College of Life Science, Sichuan Agricultural University, Yaan, China

**Keywords:** acute endometritis, intestinal microbiota, metabolomics, lipopolysaccharide, mice

## Abstract

Endometritis is generally caused by bacterial infections, including both acute and chronic infections. In the past few decades, accumulated evidence showed that the occurrence of diseases might be related to gut microbiota. The progression of diseases is previously known to change the composition and diversity of intestinal microbiota. Additionally, it also causes corresponding changes in metabolites, primarily by affecting the physiological processes of microbiota. However, the effects of acute endometritis on intestinal microbiota and its metabolism remain unknown. Thus, the present study aimed to assess the effects of acute endometritis on intestinal microbes and their metabolites. Briefly, endometritis was induced in 30 specific pathogen-free (SPF) BALB/c female mice *via* intrauterine administration of lipopolysaccharide (LPS) after anesthesia. Following this, 16S rRNA gene sequencing and liquid chromatogram-mass spectrometry (LC-MS) were performed. At the genus level, the relative abundance of *Klebsiella*, *Lachnoclostridium_5*, and *Citrobacter* was found to be greater in the LPS group than in the control group. Importantly, the control group exhibited a higher ratio of *Christensenellaceae_R−7_group* and *Parasutterella*. Furthermore, intestinal metabolomics analysis in mice showed that acute endometritis altered the concentration of intestinal metabolites and affected biological oxidation, energy metabolism, and biosynthesis of primary bile acids. The correlation analysis between microbial diversity and metabolome provided a basis for a comprehensive understanding of the composition and function of the microbial community. Altogether, the findings of this study would be helpful in the prevention and treatment of acute endometritis in the future.

## Introduction

Endometritis is a disease caused by bacterial pathogens, such as Chlamydia trachomatis, Enterococcus, Escherichia coli, Gardnerella vaginalis, Klebsiella pneumoniae, Mycoplasma hominis, Neisseria gonorrhoeae, Staphylococcus, and Streptococcus (Moreno et al., 2018). These bacterial pathogens are known to cause persistent inflammation of the uterus, which could further result in infertility in severe cases (Wallach and Czernobilsky, 1978; Kitaya et al., 2018). Among these, E. coli and Staphylococcus aureus are particularly recognized as important factors in the infection of endometritis (Andrews et al., 2006; Kitaya et al., 2017). Importantly, lipopolysaccharide (LPS) produced by E. coli is considered to be the triggering factor for various inflammatory reactions, which play a vital role in the establishment of inflammatory models (Chen et al., 2010; Wu et al., 2016; Wang and Zhang, 2018).

Throughout long-term evolution, the mammalian intestines have evolved into a complex environment. The intestines usually consist of a dense microbiome that is strongly associated with host health and disease status (Marchesi et al., 2016). Intestinal microbiota not only affects normal metabolism (Sharon et al., 2014) but also regulates host immunity (Rooks and Garrett, 2016), nervous system (Tan et al., 2020), and even cancer development (Yachida et al., 2019). In particular, it acts as a barrier against pathogen invasion, protects the intestinal structure, and maintains normal physiological function. In fact, any imbalance in the gut microbiome can cause corresponding physiological effects. Previous studies have shown that imbalances in the gut microbiota could lead to an increase in estrogen secretion (Plottel and Blaser, 2011), which was associated with endometriosis, endometrial cancer, and other uterine diseases (Borella et al., 2021). However, its role in endometritis remains poorly understood.

The microbiome represents a dynamic community, whose composition is mainly influenced by age, disease status, eating habits, and other factors. These factors influence the composition and diversity of microorganisms. Additionally, these also affect the physiological processes of microorganisms, such that the distribution of their metabolites also changes. Variations in the microbial metabolic spectrum could further cause physiological changes in both host and pathogenic microorganisms, thereby affecting the progression of the disease (Cameron and Sperandio, 2015). Consequently, metabolism studies and metabolic profiling are used as markers for the diagnosis of diseases (Ursell et al., 2014). In particular, plasma trimethylamine nitrogen oxide (TMAO) has been previously identified as a marker for cardiovascular disease (CVD) (Koeth et al., 2013), whereas 3-carboxy-4-methyl-5-propyl-2-furanpropionic acid (CMPF) was identified in the plasma of patients with gestational diabetes, impaired glucose tolerance, and type 2 diabetes (Prentice et al., 2014). However, no previous studies reported the identification of metabolites associated with endometritis.

The present study aimed to assess the effects of acute endometritis on intestinal microbes and their metabolites. In particular, it was hypothesized that administration of lipopolysaccharide (LPS) would induce endometritis and affect the intestinal microbiota and metabolites in the mice model. Herein, the intestinal tissues of acute endometritis mice were subjected to 16S rRNA high-throughput sequencing technology and liquid chromatogram-mass spectrometry (LC-MS) non-targeted metabolism technology, to assess the distribution of intestinal metabolites and unravel the composition of microbiota present in the mice with acute endometritis. Altogether, the results of the study would provide possible molecular markers for the diagnosis of acute endometritis.

## Materials and Methods

### Animals

All experimental procedures, including animal handling, welfare monitoring, and euthanasia, were performed following the ARRIVE guidelines and regulations and were approved by the Animal Care Office of Chengdu Normal University, Chengdu, China. Specific pathogen-free (SPF) BALB/c female mice aged 6–8 weeks were purchased from the Chengdu Dossy Experimental Animals Co., Ltd.

### Experimental Processing

A total of 30 mice were placed at a temperature of 25 ± 3°C, under 75 ± 5% humidity, fixed with 12-h light/12-h dark treatment daily, and provided adequate food and water. The mice were randomly categorized into five groups: the LPS group (3, 6, 12, and 24 h) and the control group. The murine model of endometritis was established as previously described (Wu et al., 2016; Wu et al., 2018). Briefly, each side of the mouse uterus was perfused with 20 μl of LPS (3 mg/ml) under anesthesia. The LPS was brought from Sigma-Aldrich (USA). The control group mice were intraperitoneally injected with the same amount of phosphate-buffered saline (PBS; Beijing Labgic Technology Co., Beijing, China). The uterine and intestinal tissues were collected at 3, 6, 12, and 24 h. The samples were frozen under liquid nitrogen immediately after collection at −80°C.

### Inflammation Analysis

The uterine tissues were fixed with paraformaldehyde and then pruned, dehydrated, and paraffin-embedded. Five sections were stained with hematoxylin and eosin (H&E) before microscopic observation (Nikon, Eclipse Ci-L, Japan). The expressions of interleukin (IL)-6, IL-1β, and tumor-necrosis factor-alpha (TNF-α) were detected by quantitative real-time polymerase chain reaction (PCR). Total RNA was extracted from the uterine tissues by using the MiniBEST Universal RNA Extraction Kit (Takara, Japan) and reverse transcribed into cDNA. The cDNA product was diluted with the Fast qPCR Master Mix (High Rox, BBI, ABI) on the StepOne Plus Fluorescent Quantitative PCR instrument (ABI, Foster, CA, USA). Primers (listed in **Supplementary Table 1**) were designed using the Primer Premier 5.0 software, and the relative quantification of the target gene expression was performed using the 2^−ΔΔCt^ method. Statistical analyses were performed using the GraphPad Prism 8 (GraphPad InStat Software, USA). Comparison between the groups was performed using *t*-test, and the data were expressed as mean ± SD. p < 0.05 was considered to indicate statistical significance.

### DNA Extraction and Library Construction

Total genomic DNA was extracted according to the instructions for the QIAamp 9 PowerFecalQIAcube HT Kit (Qiagen, 51531). The concentration of DNA was verified by the Nano Drop system (Thermo Fisher, 2000) and agarose gel. Using the genomic DNA as a template, according to the selection of sequencing V3–V4 variable regions [primers 343F: 5′-TACGGRAGGCAGCAG-3′, 798R: 5′-AGGGTATCTAATCCT-3′ (Nossa et al., 2010)], TksGflex DNA polymerase (Takara, R060B) and specific primers with barcode were used for PCR. The quality of amplifiers was confirmed by gel electrophoresis, purified by the AMPoule XP Bead (Agencourt), followed by another round of PCR amplification. The Qubit dsDNA Analysis Kit (Life Technologies, Q32854) was used to quantify the final amplifier after purification of the ampoule XP bead. An equal number of purified amplifiers were assembled for subsequent sequencing.

### Analysis of Intestinal Microbiota

The Trimmomatic software was used to preprocess the paired-end reads (Bolger et al., 2014), pruned, and assembled by the FLASH software after trimming (Reyon et al., 2012). The assembly parameters were as follows: minimum overlap, 10 bp; maximum overlap, 200 bp; and maximum error ratio, 20%. Abandoned homologous sequences are those <200 bp; 75% of the base readings above Q20 were retained. The chimera readings were detected and removed by using the UCHIME (Caporaso et al., 2010). Vsearch software was used to generate operational taxonomic units (OTU) by removing the primer sequences and clustering with a cutoff value of 97% similarity (Rognes et al., 2016). The representative reading of each OTU was selected using the QIIME package. The RDP classifier was used to annotate the species of all representative reads according to the Silva database (version 123) (confidence threshold 70%) (Wang et al., 2007).

### Metabolomics Processing

We accurately weighed 15 mg of the tissue samples into 1.5-ml of the EP tube and added the inner standard (FMOC-L-2-Chlorophe, 0.3 mg/ml; Lyso PC17: 0, 0.01 mg/ml, all configured with methanol) of 20 μl and added 400 μl of methanol–water (v/v = 4:1). After grinding, centrifugation, supernatant absorption, filtration, and transfer to the LC sample vial, the solution was stored at −80°C until LC-MS analysis.

For data processing, the metabolic profiling in positive and negative electrospray ionization (ESI) modes was analyzed by using the liquid-mass spectrometry system consisting of the Dionex U3000 UHPLC High-Resolution Mass Spectrometer and the QE plus (Thermo Fisher Scientific, Waltham, MA, USA). The determination was performed on the ACQUITY UPLC HSS T3 (100 × 2.1 mm, 1.8 μm) with a mobile phase consisting of A-water (containing 0.1% formic acid, v/v) and B-acetonitrile (containing 0.1% formic acid, v/v). The flowrate was set to 0.35 ml/min, and the column temperature was 45°C. The injection volume was 2 µl. Data acquisition was performed in the full-scan mode (m/z ranges from 70 to 1,000) combined with the IDA mode.

### Metabolomics Data Analysis

The Progenesis Qi V2.3 software (Nonlinear Dynamics, Newcastle, UK) was used to process the metabolic raw data after collection by Unifi 1.8.1. The compounds were identified based on the accurate mass number, secondary fragments, and isotope distribution using The Human Metabolome Database (HMDB), Lipidmaps (V2.3), METLIN databases, and self-built databases. The qualitative compounds were screened according to the qualitative results score of the compounds. The screening standard was 36 points (full score = 60 points), and the qualitative results <36 points were considered to be inaccurate and deleted.

Principle component analysis (PCA) and (orthogonal) partial least-square-discriminant analysis (O) PLS-DA were performed to observe the overall metabolic differences among the groups. The Hotelling’s T2 region demonstrated an ellipse in the model score, which was defined at a 95% confidence interval for model variation. In the OPLS-DA analysis, variable importance in the projection (VIP) was employed to measure the influence and explanatory ability of the samples in each group; VIP >1 was regarded as the screening criteria. We selected differential metabolites according to the threshold of statistically significant variables obtained from the OPLS-DA model on (VIP) values and p-values obtained from two-tailed Student’s *t*-test of normalized peak areas. Metabolites with VIP values >1.0 and p < 0.05 were considered to indicate differential metabolites.

## Results

### Effect of LPS on Inflammation of Mouse Uterus

**Figure 1:**
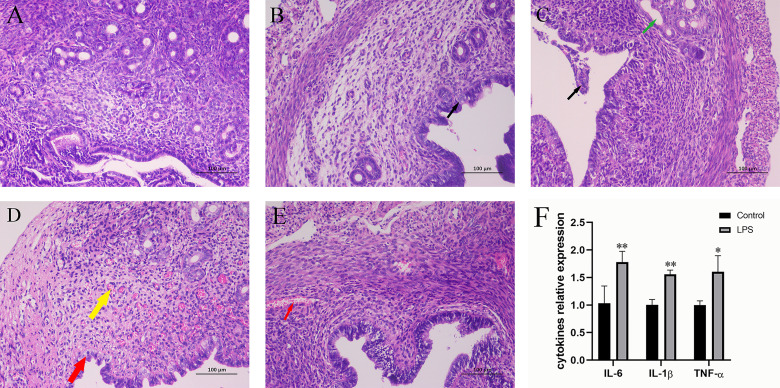
Effect of LPS on inflammation of the mouse uterus. **(A)** Control group. **(B)** LPS group (3 h). A small amount of endometrial epithelial cells seems swollen, and the cytoplasm is loose and light-stained (black arrow). **(C)** LPS group (6 h). Bits of endometrial epithelial cells are shed (black arrow), and a small number of uterine glands are slightly dilated (green arrow). **(D)** LPS group (12 h). The endometrial epithelium and glandular epithelium are swollen, the cytoplasm has loosened and lightly stained (red arrow), and a large number of capillaries in the lamina propriety are congested and dilated (yellow arrow). **(E)** LPS group (24 h). A spot of blood stasis in the lamina propria (red arrow). (Hematoxylin and eosin staining; magnification, 200×). **(F)** The expression of inflammatory cytokines IL-6, IL-1β, and TNF-α. Mean ± SD was employed for data processing. Three replicates were processed in each group. ^*^p < 0.05, ^**^p < 0.01 vs. control group.

The effect of LPS on uterine inflammation in mice was assessed by evaluating histopathological conditions. As shown in **Figures 1A**–**E**, the histopathological assessment showed that the morphology of uterine tissue was normal in the control group. An increase in LPS treatment time resulted in a gradual enhancement of the pathological manifestations, and the pathological situation was reported to be most serious at 12 h. After 12 h, the pathological condition of uterine tissue weakened. In addition to this, changes in cytokine-related expression levels were also observed (**Figure 1F**). In fact, LPS treatment for 12 h significantly increased the levels of IL-6, IL-1β, and TNF‐α.

### Effects of LPS on Diversity, Richness, and Composition of Intestinal Microorganisms in Mice

**Figure 2:**
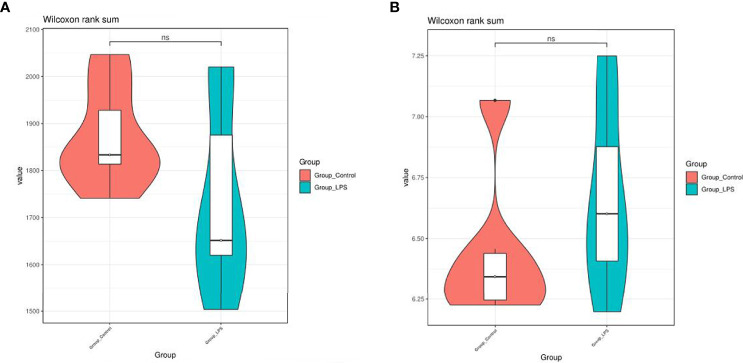
Effects of LPS on the diversity and richness of intestinal microbiota in mice. **(A)** Chao1 index. **(B)** Shannon index. ns means no significant difference.

Following quality controlled processing of the original sequencing results obtained from 16S rRNA sequencing, the data volume obtained for clean tags was distributed between 89734–93578. After removing chimeras, the valid tags obtained for analysis were allocated between 80742 and 85484. Valid tags were divided into OTU according to 97% similarity, and a total of 4,373 OTUs were obtained. According to the results of the Shannon index and Chao1 index (**Figure 2**), no significant differences were recorded between the diversity of gut microbiome in mice treated with LPS and normal mice.

**Figure 3:**
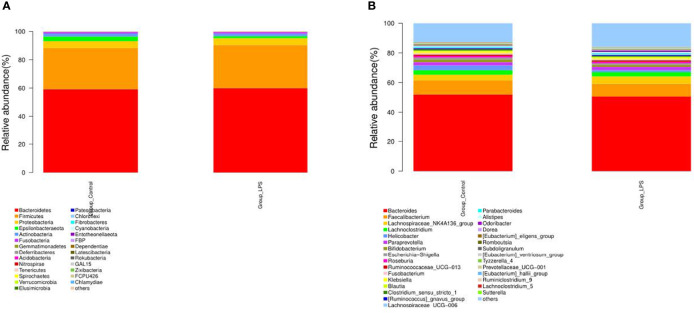
Effect of LPS on the composition of intestinal microbiota in mice. **(A)** Phylum level. **(B)** Genus level.

**Figure 4:**
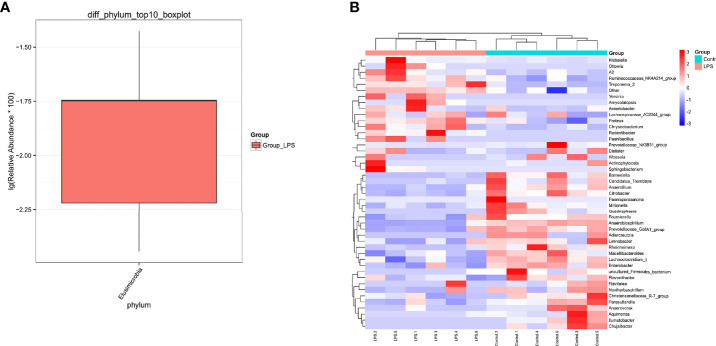
Differential species of the intestinal microbiota of mice in the LPS group and control group. **(A)** The phylum level is evident from the box drawing. **(B)** The genus level is represented by a heat map. Orange indicates relatively high species abundance, while blue indicates relatively low species abundance.

In particular, a total of 27 bacterial phyla were detected for the classification of the resulting OTUs. Among these, *Bacteroidetes* and *Firmicutes* were found to be the dominant ones, which accounted for 59.56% and 29.86%, respectively. At the genus level, a total of 529 bacterial genera were detected. Among these, the dominant species were *Bacteroides* (51.25%), *Faecalibacterium* (9.05%), *Lachnospiraceae_NK4A136_group* (4.07%), *Lachnoclostridium* (2.99%), *Helicobacter* (2.66%), and *Paraprevotella* (2.02%) (**Figure 3**). In addition to this, the study also analyzed different species present in each group using the Wilcoxon test (**Figure 4**). At the phylum level, *Elusimicrobia* was identified as a distinct phylum. Furthermore, at the generic level, *Klebsiella*, *Lachnoclostridium_5*, *Citrobacter*, *Enterobacter*, *Treponema_2*, *Christensenellaceae_R−7_group*, and *Parasutterella* were found to be different in the two groups.

### Effects of LPS on Intestinal Metabolomics in Mice

**Figure 5:**
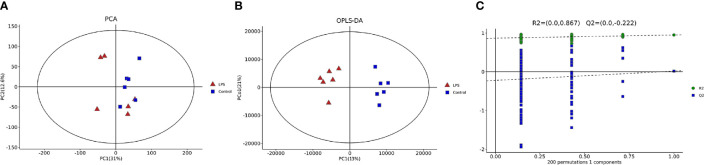
Multivariate statistical analyses of intestinal metabolites in mice. **(A)** Principal component analysis (PCA). **(B)** Orthogonal partial least-squares-discriminant analysis (OPLS-DA) diagram. **(C)** Validation diagram obtained from the permutation test.

LC-MS non-targeted metabolomics analysis was conducted for the intestinal samples of the control group and LPS group. The principal component analysis showed a separation between the samples obtained from these two groups (**Figure 5A**). OPLS-DA was utilized to verify differential metabolites between the two groups, and multivariate analysis was supervised. Furthermore, the score plot showed/revealed significant differences in OPLS-DA score for the two groups of samples (**Figure 5B**). As shown in **Figure 5C**, the OPLS-DA fitting model did not show overfitting of the model. Meanwhile, for the displacement test, the values for R2Y (0.867) and Q2Y (−0.222 < 0) also indicated the validity of the model.

The study also compared the metabolites (**Supplementary Table 2**) between the two groups, by *t*-test and multivariate analysis combined with variable influence on projection (VIP). A total of 187 different metabolites were identified (**Supplementary Table 3**). Among the 76 positive ion metabolites, two were identified as benzenoids, 23 were lipids and function‐like molecules, and one belonged to nucleosides, nucleotides, and analogs. Additionally, these positive ion metabolites included nine kinds of organic acids and derivatives, four types of organic oxygen compounds, four types of organoheterocyclic compounds, one organosulfur compound, and two phenylpropanoids and polyketides. In the case of negative‐ion metabolites, a total of 111 such metabolites were identified, which were divided into alkaloids and derivatives, benzenoids, lipids, and lipid‐like molecules, nucleosides, nucleotides, and their analogs, organic acids and derivatives, organic compounds, organic oxygen compounds, organoheterocyclic compounds, phenylpropanoids and polyketides, and others.

**Figure 6:**
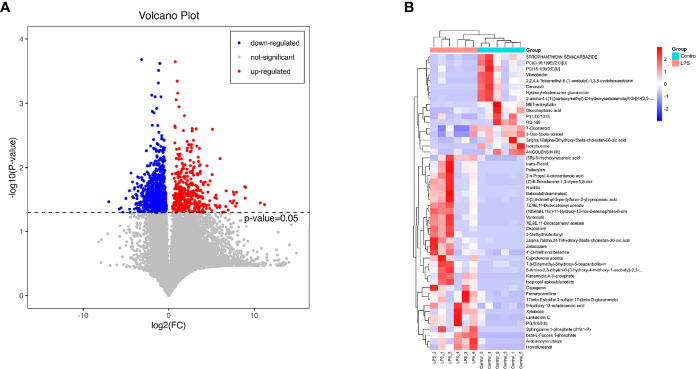
Effects of LPS on the intestinal metabolites in mice. **(A)** Significantly different metabolites, in which the red dots represent significantly upregulated differential metabolites in the LPS group, the blue dots represent significantly downregulated differential metabolites, and the gray dots represent insignificant differential metabolites. **(B)** HCA. The abscissa expresses the sample name, and the ordinate represents the differential metabolites. The color ranges from blue to red, indicating the expression abundance of metabolites from minimal to high, that is, the greater the intensity of the red color, the higher is the expression abundance of differential metabolites.

**Figure 7:**
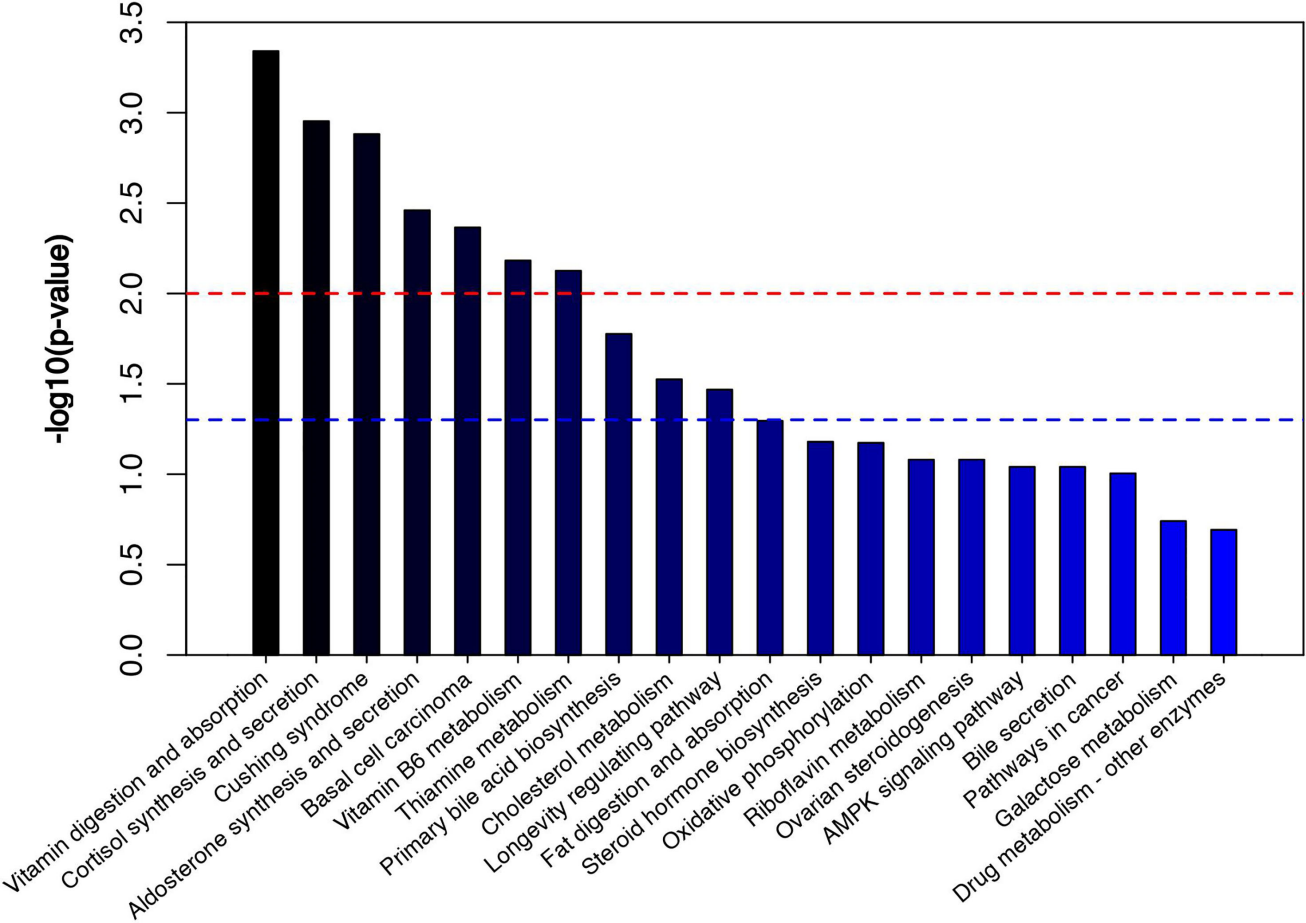
Metabolic pathway enrichment diagram. The red line indicates p = 0.01, while the blue line indicates p = 0.05. When the top of the bar is higher than that of the blue line, the signal pathway represented is significant.

The volcano diagram showed dramatic up- and downregulation of metabolites in the endometritis group and normal group (**Figure 6A**). To display the relationship between samples and differences in the expression of metabolites among different samples, hierarchical clustering analysis (HCA) was performed on the expression levels of top 50 significantly different metabolites, and the clustering results were shown in terms of a heat map (**Figure 6B**). In the positive ion mode, LPS treatment increased the expression of metabolites, such as cholesterol, 11‐deoxycortisol, and N‐phenylacetylphenylalanine, and reduced the expression of Darunavir, PE (19:1(9Z)/0:0), and LysoPE (24:1(15Z)/0:0). However, in the negative ion mode, LPS treatment resulted in an increase in metabolites, like trans-piceid and Psilocybin, and decreased the levels of NAD, MET‐enkephalin, and Isokobusone. In addition to this, KEGG ID for differential metabolites was used for pathway enrichment analysis (**Figure 7**). In particular, the metabolic pathways for cholesterol metabolism, primary bile acids biosynthesis, and vitamin digestion and absorption were markedly affected by LPS.

**Figure 8:**
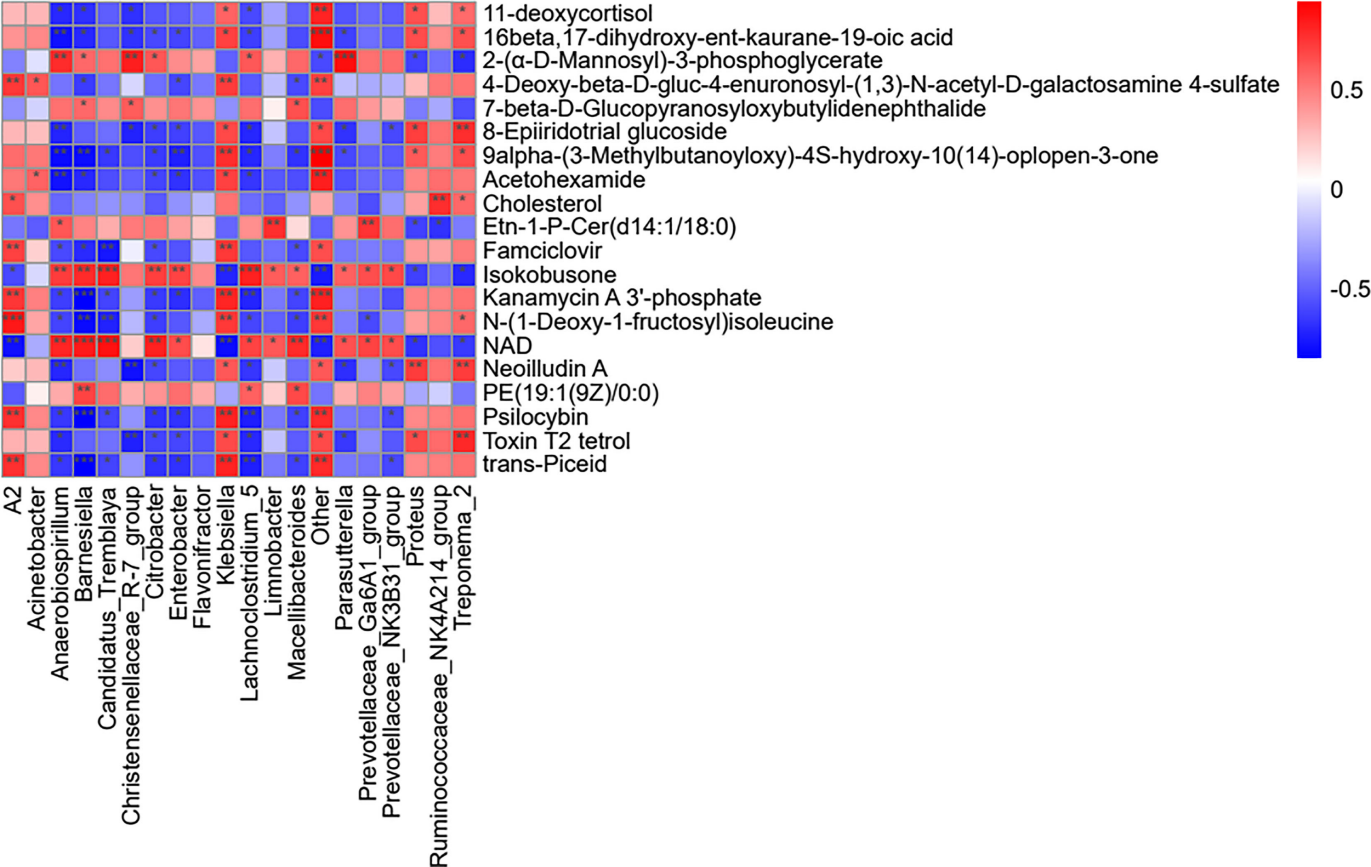
Analysis of the effects of LPS on the microbial genus and metabolite concentration. For each species with distinct behaviors and the corresponding metabolites in each column, the orange color indicates a positive correlation, while blue indicates a negative correlation. The darker the color, the greater is the correlation. The closer the color is to white, the closer is the correlation to zero. ***p < 0.001, **p < 0.01, and *p < 0.05 (i.e., significance of correlation).

Importantly, changes in the metabolic spectrum of the microbiome reflect changes in the dynamics of the microbiome. Therefore, the present study analyzed the correlation between microbial diversity and metabolomics to understand the composition and function of microorganisms in a better way. As shown in **Figure 8**, *Treponema_2* positively correlated with the levels of cholesterol in the microbial community, with higher genera expression abundance in the LPS group, and negatively correlated with NAD. In the normal control group, *Parasutterella* was found to be positively correlated with NAD and Isokobusone and negatively correlated with 8-Epiiridotrial glucoside in the microbial community, with superior generic-level expression abundance.

## Discussion

As a bacterial infectious disease, endometritis is known to primarily affect the life of women and modern agricultural production, which is detrimental to human health and economic development (Zhao et al., 2017). Lipopolysaccharide (LPS) obtained from the cell wall of Gram-negative bacteria has been previously shown to play an important role in the pathogenesis of endometritis (Li et al., 2019; Zhou et al., 2019). Thus, it is used to induce endometritis in mice. Currently, the gold standard used for endometritis is histopathological examination (Kiviat et al., 1990; Kitaya and Yasuo, 2011). In the present study, H&E staining of the tissue samples and assessment of tissue morphology were used for the evaluation of the tissue samples. Briefly, after 12 h of LPS treatment, endometrium and uterine glandular epithelial cells were found to be swollen, the cytoplasm was loose and lightly stained, and a large number of capillaries in lamina propriety stasis and dilation were recorded as the most serious pathological conditions. Consequently, the tissues treated with LPS for 12 h were used for subsequent analysis. Since LPS can induce the production of cytokines, such as IL-6, IL-1β, and TNF‐α (Ahmad et al., 2019), the present study also assessed the related expression levels of cytokines in uterine tissues at 12 h, after LPS induction. The results showed that LPS treatment for 12 h significantly increased the levels of IL-6, IL-1β, and TNF‐α, indicating the successful establishment of the LPS-induced endometritis model in mice.

The occurrence of diseases has been previously shown to cause changes in the gut microbiota and its metabolism (Rooks and Garrett, 2016). No previous studies investigated the relation between acute endometritis and gut microbiota. The present study reported changes in intestinal microbiome and metabolism of mice with acute endometritis, using 16SrRNA high-throughput sequencing and LC-MS technology. The study also reported differences in the composition and metabolites of the microbiome structure and metabolites present in the intestines of mice with acute endometritis, induced by LPS. *Elusimicrobia* is known to exert a potential negative impact on health, and it is often used as a biomarker for intestinal damage (Carbonero et al., 2019). At the genus level, a decrease in beneficial bacteria, such as *Christensenellaceae_R.7_group* and *Parasutterella*, was recorded in the LPS group in the present case. An increase in the relative abundance of *Treponema_2*, *Klebsiella*, *Lachnoclostridium_5*, and *Citrobacter* was also recorded. In general, *Christensenellaceae_R.7_Group* is a microorganism that is widely present in humans and animals. It is primarily associated with obesity and inflammatory bowel disease (Waters and Ley, 2019). The relative abundance of *Christensenellaceae_R.7_group* has been reported to be comparatively low in obese patients (Valdes et al., 2018). However, obese people generally exhibit a higher risk of endometritis as compared to the general population (Kalkanbaeva et al., 2017). *Parasutterella* is an important microorganism that maintains the health of the human gastrointestinal tract. It is associated with many diseases (Blasco-Baque et al., 2017; Ju et al., 2019). In comparison to this, *Klebsiella* is a pathogen that primarily causes severe suppurative community-acquired pneumonia, which can infect almost any part of the body and is extremely lethal (Sahly et al., 2002). *Lachnoclostridium_5* is usually associated with digestive diseases (Wang et al., 2018). *Citrobacter* is an extracellular intestinal pathogen that is specifically designed to mimic human pathogenic *E. coli* and inflammatory bowel disease infections (Mullineaux-Sanders et al., 2019). In the present study, these bacteria genera exhibited differential expression in the LPS group, which indicated that after acute endometritis, the abundance of intestinal pathogenic bacteria increased, while the beneficial bacteria decreased, and the protection provided by intestinal barrier also reduced. Consequently, the possibility of the disease increased.

In addition to this, the amount of intestinal metabolite cholesterol was recorded to be higher in the LPS group. Cholesterol is known to be an essential molecule in the animal body, which participates in various biochemical processes, such as cell membrane synthesis and cell growth and differentiation (Russell, 1992). However, the inadequate ability of animals to decompose cholesterol can lead to an increase in the level of cholesterol that can further result in diseases, such as atherosclerosis (Schade et al., 2020). Besides this, acute endometritis also resulted in reduced levels of NAD and Isokobusone. Nicotinamide adenine dinucleotide (NAD) is known for its role in redox reactions, particularly as a hydrogen carrier for cellular oxidoreductases. It also acts as a signaling molecule that regulates hundreds of key processes, including energy metabolism and cell survival, *via* regulation of NAD^+^ sensing enzymes. NAD^+^ levels usually decline with age, resulting in metabolic changes and increased susceptibility to disease (Rajman et al., 2018). A previous study reported that Isokobusone could activate pregnane X receptor (PXR) and constitutive androstane receptor (CAR) and thus induced the expression of drug metabolism enzymes and inhibited the expression of LPS‐induced inflammatory mediators (Kittayaruksakul et al., 2013). Therefore, acute endometritis altered the levels of metabolites in the body, which further affected normal biological redox responses, decreased the inhibition of inflammatory response, and increased susceptibility to the disease.

The combination of microbiology and metabolomics revealed the changes in the composition of the intestinal microbiome in acute endometritis, which suggested that the activity of the gut microbiome might be related to intestinal metabolism. It was found that the abnormal expression of *Treponema_2* in the LPS group positively correlated with cholesterol, wherein Treponema cells could obtain cholesterol from the erythrocytic membranes of eukaryotes (Stanton and Cornell, 1987) and produced exogenous cholesterols during growth. Cholesterol depletion or incorporation of cholesterol molecules might harm the intestinal epithelial membrane. In addition to this, acute endometritis also decreased the relative abundance of *Parasutterella*. *Parasutterella* has been previously shown to be associated with bile acid homeostasis (Ju et al., 2019). Biosynthesis of primary bile acids is generally involved in the pathogenesis of cervicitis. Cervicitis is known to provoke/induce a variety of diseases, including endometritis (Zhang et al., 2018). In addition to this, enrichment analysis of metabolic pathways revealed that LPS could alter the biosynthesis of primary bile acids, indicating an important role in bile acid metabolism. However, the specific mechanism of action remains to be explored in the future. In addition to this, it needs to be explored whether the altered gut microbiota and its metabolites also affected the internal environment of the uterus.

Altogether, the present study reported the induction of acute endometritis in mice upon LPS treatment, and changes were reported in intestinal microbiota and metabolites by 16S rRNA high-throughput sequencing and LC-MS untargeted metabolism. At the generic level, acute endometritis resulted in the reduction in beneficial microorganisms in the intestinal tract. At the same time, it increased the relative abundance of pathogenic bacteria, altered the metabolic levels of cholesterol and NAD, and affected the corresponding biological REDOX reactions and other biochemical processes. Thus, the findings of this study would provide new strategies for the diagnosis of acute endometritis.

## Data Availability Statement

The data presented in the study are deposited in the Sequence Read Archive of the National Center for Biotechnology Information repository, accession number PRJNA782690.

## Ethics Statement

The animal study was reviewed and approved by Animal Care Office of Chengdu Normal University, Chengdu, China.

## Author Contributions

Conceptualization: YD. Methodology: YY, YM, YL, and WZ. Formal analysis: YD, YY. Investigation: XD and JP. Validation: YY and YD. Data curation: BH and YD. Writing—original draft preparation: YD and YY. Writing—review and editing: YM, BH, and SL. Funding acquisition: BH and SL. All authors contributed to the article and approved the submitted version.

## Funding

This work was supported by the Chengdu Normal University scientific research project (No. 111-153701) and the Agricultural Ecology and Green Food Development project (CSCXTD2020B11). The funders had no role in the study design, data collection, and analysis, decision to publish, or preparation of the manuscript.

## Conflict of Interest

The authors declare that the research was conducted in the absence of any commercial or financial relationships that could be construed as a potential conflict of interest.

## Publisher’s Note

All claims expressed in this article are solely those of the authors and do not necessarily represent those of their affiliated organizations, or those of the publisher, the editors and the reviewers. Any product that may be evaluated in this article, or claim that may be made by its manufacturer, is not guaranteed or endorsed by the publisher.
