# Corrigendum: Combined intestinal metabolomics and microbiota analysis for acute endometritis induced by lipopolysaccharide in mice

**DOI:** 10.3389/fcimb.2023.1223663

**Published:** 2023-06-09

**Authors:** Yuqing Dong, Yuan Yuan, Yichuan Ma, Yuanyue Luo, Wenjing Zhou, Xin Deng, Jingyu Pu, Binhong Hu, Songqing Liu

**Affiliations:** ^1^ College of Chemistry and Life Sciences, Chengdu Normal University, Chengdu, China; ^2^ College of Forestry, Sichuan Agricultural University, Chengdu, China; ^3^ College of Life Science, Sichuan Agricultural University, Yaan, China

**Keywords:** acute endometritis, intestinal microbiota, metabolomics, lipopolysaccharide, mice

**Error in Figure/Table**

In the published article, there was an error in **Figure 1** as published. This Figure was used incorrectly. The corrected **Figure 1** and its caption appear below.

**Figure 1:**
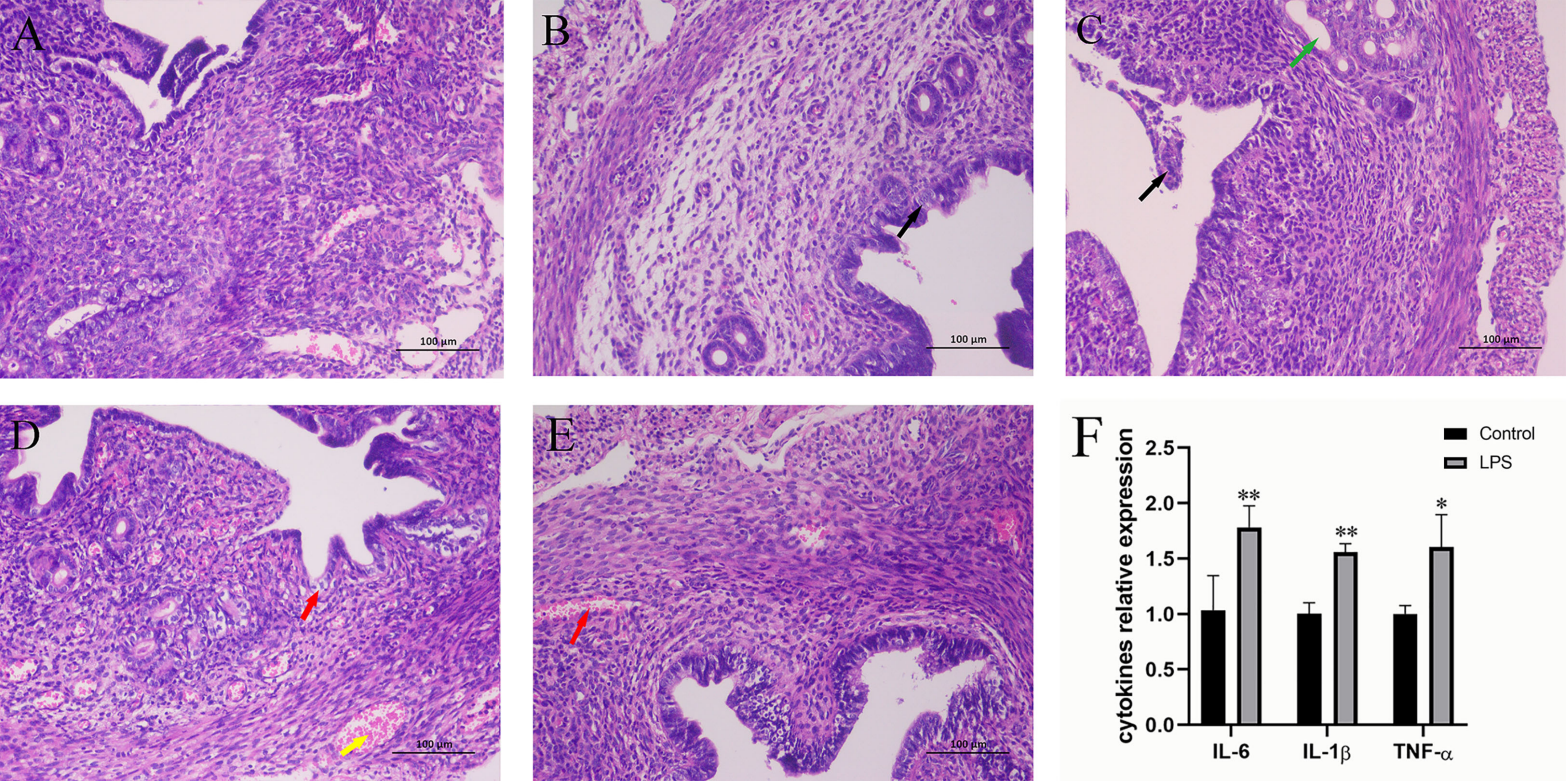
Effect of LPS on inflammation of the mouse uterus. **(A)** Control group. **(B)** LPS group (3 h). A small amount of endometrial epithelial cells seems swollen, and the cytoplasm is loose and light-stained (black arrow). **(C)** LPS group (6 h). Bits of endometrial epithelial cells are shed (black arrow), and a small number of uterine glands are slightly dilated (green arrow). **(D)** LPS group (12 h). The endometrial epithelium and glandular epithelium are swollen, the cytoplasm has loosened and lightly stained (red arrow), and a large number of capillaries in the lamina propriety are congested and dilated (yellow arrow). **(E)** LPS group (24 h). A spot of blood stasis in the lamina propria (red arrow). (Hematoxylin and eosin staining; magnification, 200×). **(F)** The expression of inflammatory cytokines IL-6, IL-1β, and TNF-α. Mean ± SD was employed for data processing. Three replicates were processed in each group. *p < 0.05, **p < 0.01 vs. control group.

Caption: Effect of LPS on inflammation of the mouse uterus. (A) Control group. (B) LPS group (3 h). A small amount of endometrial epithelial cells seems swollen, and the cytoplasm is loose and light-stained (black arrow). (C) LPS group (6 h). Bits of endometrial epithelial cells are shed (black arrow), and a small number of uterine glands are slightly dilated (green arrow). (D) LPS group (12 h). The endometrial epithelium and glandular epithelium are swollen, the cytoplasm has loosened and lightly stained (red arrow), and a large number of capillaries in the lamina propriety are congested and dilated (yellow arrow). (E) LPS group (24 h). A spot of blood stasis in the lamina propria (red arrow). (Hematoxylin and eosin staining; magnification, 200×). (F) The expression of inflammatory cytokines IL-6, IL-1β, and TNF-α. Mean ± SD was employed for data processing. Three replicates were processed in each group. *p < 0.05, **p < 0.01 vs. control group.

The authors apologize for this error and state that this does not change the scientific conclusions of the article in any way. The original article has been updated.

